# Different strategies do not moderate primary motor cortex involvement in mental rotation: a TMS study

**DOI:** 10.1186/1744-9081-3-38

**Published:** 2007-08-07

**Authors:** Stefan Bode, Susan Koeneke, Lutz Jäncke

**Affiliations:** 1 Max-Planck-Institute for Human and Cognitive Brain Sciences, Leipzig, Germany; 2 University of Zurich, Institute of Psychology, Division Neuropsychology, Switzerland

## Abstract

**Background:**

Regions of the dorsal visual stream are known to play an essential role during the process of mental rotation. The functional role of the primary motor cortex (M1) in mental rotation is however less clear. It has been suggested that the strategy used to mentally rotate objects determines M1 involvement. Based on the strategy hypothesis that distinguishes between an internal and an external strategy, our study was designed to specifically test the relation between strategy and M1 activity.

**Methods:**

Twenty-two subjects were asked to participate in a standard mental rotation task. We used specific picture stimuli that were supposed to trigger either the internal (e.g. pictures of hands or tools) or the external strategy (e.g. pictures of houses or abstract figures). The strategy hypothesis predicts an involvement of M1 only in case of stimuli triggering the internal strategy (imagine grasping and rotating the object by oneself). Single-pulse Transcranial Magnetic Stimulation (TMS) was employed to quantify M1 activity during task performance by measuring Motor Evoked Potentials (MEPs) at the right hand muscle.

**Results:**

Contrary to the strategy hypothesis, we found no interaction between stimulus category and corticospinal excitability. Instead, corticospinal excitability was generally increased compared with a resting baseline although subjects indicated more frequent use of the external strategy for all object categories.

**Conclusion:**

This finding suggests that M1 involvement is not exclusively linked with the use of the internal strategy but rather directly with the process of mental rotation. Alternatively, our results might support the hypothesis that M1 is active due to a 'spill-over' effect from adjacent brain regions.

## Background

The ability to create and manipulate mental images is a very important psychological function in human cognition. One common approach to the study of this function was introduced by Shepard and Metzler [1]. In their studies, subjects were required to compare pairs of three-dimensional objects presented at different degrees of rotation within the picture plane and to judge as fast as possible whether the objects are identical or mirror images. The initial results point to a positive linear relationship between the degree of angular disparity between the objects and reaction time. Based on this finding, it has been argued that the mental manipulation of objects follows laws similar to those for the real manipulation of physical objects. In more recent years, brain regions and circuits involved in mental rotation have been studied intensively [2-10]. These studies show that areas of the dorsal visual stream, including visual, parietal and premotor cortex, are primarily activated. Findings regarding the involvement of the primary motor cortex (M1) are however rather inconsistent [9,11] or suggest the existence of moderating variables such as different cognitive strategies [12,3]. Kosslyn et al. [3] distinguish between two strategies: The *internal *and the *external *strategy. The *internal *strategy refers to the mental process of imagining to grasp and rotate an object with one's hands, and is therefore likely to involve M1. The *external *strategy, on the other hand, describes the imagination in front of the "inner eye" of a self-rotating object driven by an external force. In this case, M1 is not necessary for task performance and should therefore not be activated [3]. A recent brain imaging study showed that task instruction exerts substantial influence on mental rotation performance. Only subjects who were allowed to rotate a 3-D model with their hands showed M1 activation in the subsequent mental rotation task [13]. A single case study demonstrated that using cortical stimulation with an implemented electrode grid in M1 led to interference with task performance in mental rotation only when the subject was instructed to use a motor strategy similar to the internal strategy [14]. Mental rotation performance was unaffected when a visual strategy comparable to the external strategy was instructed. Besides the influence of task instruction, the *kind of stimulus *rotated is thought to be an important factor in determining the naïve use of a certain strategy [6]. Pictures of human hands, for example, have been shown to evoke M1 activation, suggesting that hand stimuli trigger the use of the *internal *strategy [12,9]. The same holds for objects that are usually grasped and manipulated in the real world, like tools. More abstract stimuli such as Shepard & Metzler figures or objects that cannot be manually rotated in the real world (e.g. houses or 2-D figures) are thought to trigger the *external *strategy because their rotation by external forces appears easier to imagine [6,9]. Recent studies using Transcranial Magnetic Stimulation (TMS) demonstrated the involvement of M1 in mental rotation of body parts (hands, feet), thus providing support for the theory outlined above. Applying single pulse TMS to the left M1 hand area either 400 ms [15] or 650 ms [16] after stimulus onset slowed response times in the mental rotation task. In order to specify the functional role of M1 in mental rotation, a study from our lab used single pulse TMS applied over the left M1 hand area during several tasks: mental rotation of Shepard & Metzler figures, reading aloud, reading silently and simple mirrored-not-mirrored judgements with Shepard & Metzler figures [17]. Motor evoked potentials (MEPs) were recorded at the Abductor Pollicis Brevis (APB) muscle of the right hand to quantify corticospinal excitability. An increase of corticospinal excitability was found during mental rotation performance only. It was therefore concluded (a) that mental rotation itself causes the increase of M1 excitability, and (b) that the use of verbal strategies cannot explain increased M1 activity. The issue of other strategy differences was not investigated in that study, but it is possible that the subjects preferred to use the internal strategy and that the imposed strategy bias may have facilitated M1 activity. Therefore, we set up an experiment that aimed to specifically explore the effect of mental rotation strategy on M1 activity. We adopted the general experimental design from Eisenegger et al. [17] in order to assure comparability, but, following Vingerhoets et al. [6], we used different categories of stimuli that are expected to trigger different strategies. Assuming that the strategy hypothesis is correct, pictures of hands and tools would implicitly trigger the use of the internal strategy and therefore cause facilitation of MEP amplitudes evoked by application of TMS over the M1 hand area. In contrast, pictures of houses, abstract 2-D figures and abstract 3-D Shepard & Metzler figures would be more likely to trigger the external strategy without involving M1. Revealing an interaction between stimulus category and MEP facilitation would therefore support the strategy hypothesis, whereas a general enhancement of MEP amplitudes independent of the stimulus category would suggest a non-specific effect of mental rotation.To avoid the chance use of the same preferential a priori strategy by the majority of our experimental sample participants, we doubled the number of subjects tested by Eisenegger et al. [17]. In addition, reaction times were recorded for each stimulus category in a second part of the experiment to control for confounds with task difficulty. We also assessed the choice of a mental rotation strategy in dependence on the different categories of stimuli using a self-constructed post-test questionnaire.

## Methods

### Subjects

22 subjects took part in this study. All subjects were classified as consistent right-handers according to the Annett Handedness Questionnaire (AHQ) [18] and the Hand Dominance Test (HDT) [19,20]. A standardized questionnaire was used to ensure that the subjects did not have a neurological, psychiatric or medical disorder. Two subjects had to be excluded while recording TMS data, one because of a very high resting motor threshold (RMT) (> 80% machine output) and the second because of difficulties with task comprehension (reporting having not understood the task during the experiment). The data of the remaining 20 subjects (11 female, mean age 26.6, SD 4.0) are reported here. Data acquisition took place at the Department of Psychology/Section Neuropsychology of the University Zurich, Switzerland, and the study was conducted in accordance with the local ethics committee and the Declaration of Helsinki (code of ethics of the world medical association). All subjects gave written approval to the test procedure after receiving detailed information about TMS and the applied procedure.

### Transcranial Magnetic Stimulation

A 'Transcranial Magnetic Stimulator' (Magstim, Whitland, Dyfed, UK) equipped with a figure-of-eight coil was used for TMS stimulation and a Sigma CX System (Sigma Medizin-Technik GmbH, Germany) for recording and analysing the Motor Evoked Potentials (MEPs). The stimuli were shown on a 21" flat screen of a Dell personal computer using the Presentation Software Package (Version 0.76, Neurobehavioral Systems).

The general experimental procedure was the same for all subjects. First, the individual resting motor threshold (RMT) was assessed. For that, the subjects were seated in a self-build comfortable chair in an upright and relaxed position. To avoid any movement that would have influenced the positioning of the stimulation, the head was fixed with a head support at the back, the front, and at one side. A construction of bracings allowed the placement of the TMS coil in every desired position. We used a figure-of-eight coil (diameter of each wing 70 mm) placed tangentially over the scalp, the handle pointing backward and rotated away from the midline by 45°. TMS single pulses were applied to the hand area of the left primary motor cortex in order to evoke a response in the contralateral Abductor Pollicis Brevis muscle (APB). An electromyogram (EMG) was recorded in order to quantify MEP amplitudes. The RMT was defined as the stimulation strength (in percent machine output) at the "hot spot" which led to EMG amplitudes above 50 μV in 50% of 10 pulses [21]. For the experiment, a stimulation intensity of 20% above the individual motor-threshold was used [16]. The optimal location for eliciting MEPs in the contralateral APB was marked on the scalp and the TMS coil was fixed exactly above this point. Subjects were asked to relax muscles completely in both hands during TMS application and task performance. The experiment consisted of two parts. In the first part, we tested for differences in MEPs elicited by single pulse TMS during mental rotation of different categories of objects. In the second part, the mental rotation procedure was repeated without TMS. Instead, reaction times were recorded to control for differences in difficulty between mental rotation of objects from different categories.

### Mental rotation and TMS

**Figure 1:**
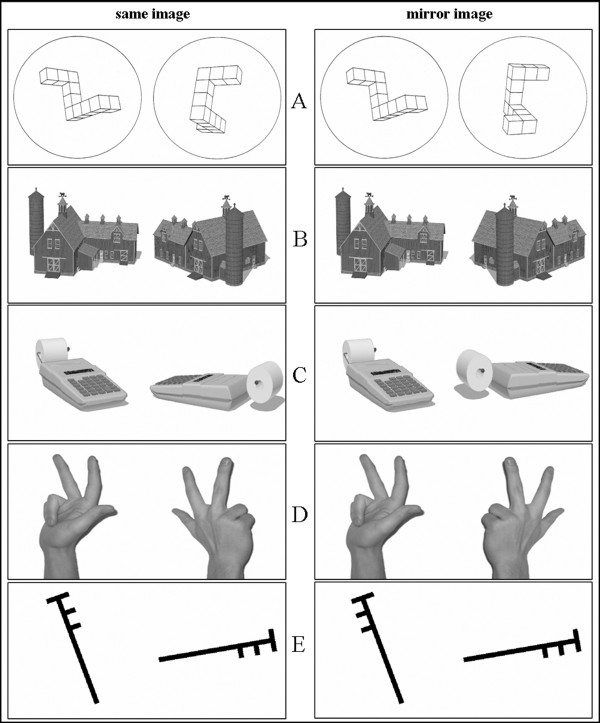
**Examples of objects pairs from the five object categories used as stimuli in the experiment**. (A) 3-D Shepard & Metzler figures (B) houses (C) tools (D) hands (E) 2-D figures. Pairs of objects shown on the left can be brought in correspondence by mental rotation ("same"). Pairs of objects on the right do not lead to a positive matching response ("mirror").

Subjects were positioned in front of the computer screen (distance = 70 ± 4 cm) on which a picture with a pair of objects appeared. Object size was kept approximately constant with a maximum of 10 cm in height or width depending on the rotation angle, resulting in a visual angle between 7.6° and 8.4° for objects from all categories (the mean visual angle did not differ between conditions). The task was to mentally rotate the left object in order to ascertain whether it could be brought into correspondence with the object image on the right. A positive match was possible in half of the trials in which the left object could be brought into correspondence with the right object by mentally rotating it. The degree of rotation necessary was different depending on the trial and ranged between 45° and 315° ("same"). In the other half of the trials, the rotation did not lead to a positive matching response, because the object on the right was a rotated mirror image of the object on the left ("mirror"). For each category, four versions (different viewing angles) of three different objects were used as stimuli for mental rotation. This ensured that subjects did not become accustomed to always seeing an object from the same viewing angle which could have otherwise led to the use of some kind of simple comparison strategy rather than mental rotation to solve the task. All stimuli were shown twice, one of the presentations allowing a matching response and the other a non-matching response. In sum, there were 24 trials in each of the five conditions (see Fig. 1): (a) 3-D Shepard & Metzler figures (b) 2-D figures of letter-like line drawings [22] (c) 3-D pictures of houses [23] (d) 3-D pictures of tools [23] and (e) 3-D pictures of hands (created from custom-made photographs, using Adobe Photoshop Version 7.0). Deviation angles between the objects were pseudo-randomized over the 24 trials. For 3-D objects, images were only rotated in the y-z plane to keep normal viewing conditions. The 2-D figures were only rotated in the picture plane. The order of conditions was pseudo-randomized between subjects and individually repeated for the second part of the experiment. Note that by using this procedure, the first learning experience was different for the subjects. Even if the first experience with a category of objects influenced the choice of strategy for the following categories [9], it should have been a different one in nearly half of the subjects.

**Figure 2:**
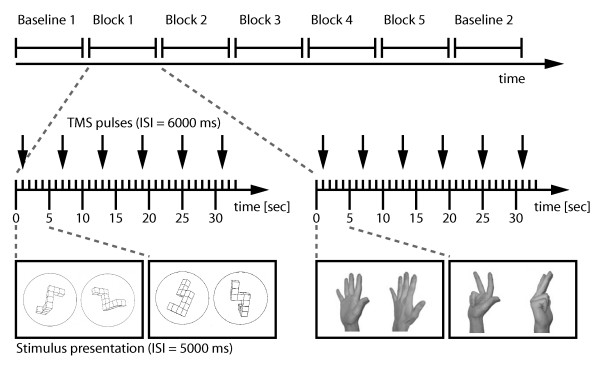
**General procedure of mental rotation and the TMS experiment**. The order of categories was pseudo-randomized for each subject (upper part). In each block objects from only one category were presented. The different pairs of stimuli were presented for 5000 ms and single pulse TMS was applied every 6000 ms as shown for the first two categories (lower part). TMS stimulation started one second after the first stimulus presentation in each block. ISI = inter-stimulus interval.

The subjects were instructed to watch every pair of objects for five seconds and to mentally rotate the left object to decide whether it was the same or a mirror image of the right object. The instructions were carefully worded in order to ensure that subjects did not adopt one particular strategy before task execution but felt free to choose their implicitly preferred strategy for every object category. To avoid any motor activity, subjects were asked not to indicate the answer (i.e. same or mirror) in any way, that is, by button press or by open or covert speech. It was important to refrain from giving any kind of response, because only this would permit enhanced excitability of M1 to be clearly linked to the mental rotation strategy itself without the confound of response-related motor activity. Instead, subjects were told to use these trials to practice for later trials in which mental rotation skills would be actually tested [17]. Additionally, they were instructed to exclusively use mental rotation to solve the task. In case subjects came to a final decision before the current trial ended, they were told to start the mental rotation operation again. A single TMS pulse was applied over the hand area of the left motor cortex every six seconds, thus covering the whole period of 24 trials with 20 pulses. MEP amplitudes were recorded in the same manner as during RMT determination. A baseline measurement preceded and followed the experimental conditions (see Fig. 2). For this, subjects were asked to relax and fixate on a cross on the screen for the same duration required for 24 trials while the same 20-pulse TMS stimulation was applied. MEP amplitudes were calculated as baseline-to-peak distances. Then, MEP amplitudes of each condition were averaged (20 MEPs per condition) and used as an indirect measure of M1 excitability during mental rotation of each category of objects and during both baseline measurements.

### Mental rotation and reaction times

In the second part of the experiment, the mental rotation procedure was repeated without TMS. Here, subjects had to respond within the five-second duration of a trial by communicating their decision by pressing one of two buttons for "same" or "mirror" with the index and middle finger of the left hand. Subjects were further instructed to perform as fast and accurate as possible. Reaction times (RTs) and errors were recorded, giving a measure of the difficulty of the conditions. Subjective difficulty was assessed after the experiment by asking the subjects to rate the conditions (mental rotation of different categories of objects) along an individual ranking of difficulty. Furthermore, they had to indicate on a five-point-Likert scale (from "0 = never used" to "4 = always used") the extent to which they used the internal strategy ("mentally rotated the objects by thinking of grasping and rotating them on my own") or the external strategy ("mentally rotated the objects by thinking of the objects rotating on their own") for each of the five conditions. Because the instructions strictly circumvented an explanation on *how *to exactly perform mental rotation, this rating permitted the assessment of the degree of naïve strategy use for objects of the different categories.

### Statistical analysis

In keeping with Eisenegger et al. [17], MEP amplitudes recorded during mental rotation (part 1 of the experiment) were analyzed using non-parametric statistics. Testing was performed using Friedman ANOVA and subsequent Wilcoxon tests, corrected for multiple comparisons. RTs were analyzed using an ANOVA for repeated measurements and post hoc Tukey tests. Parametric statistics were used here, since for RTs there was no deviation from the standard distribution in any category of objects tested by Kolmogorov-Smirnov tests (Shepard & Metzler figures: *Z *= 0.47, *p *= .98; houses: *Z *= 0.58, *p *= .89; tools: *Z *= 0.71, *p *= .70; hands: *Z *= 0.49, *p *= .97; 2-D figures: *Z *= 0.88, *p *= .42). Spearman's Rho was used for correlation analyses between MEPs and measures of difficulty like RTs, errors and ranking of difficulty. For comparisons of the number of occurrences of the different strategies (as resulting from the questionnaire), Wilcoxon tests for were performed in each object category condition separately.

## Results

**Figure 3:**
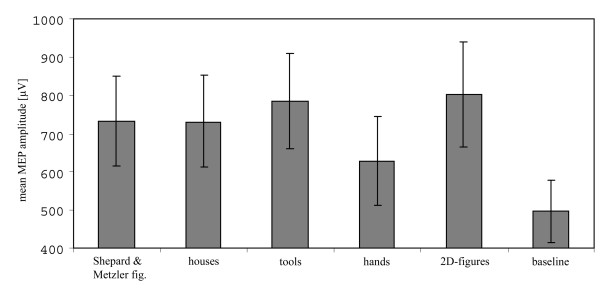
**MEP amplitudes for object categories**. Mean MEP amplitudes and standard errors (SE) recorded during mental rotation of the five object categories and for mean baseline. Differences compared with baseline were significant for all categories (p < .001), except for hands.

Because subjects' responses to mental rotation were not recorded during the TMS session, they were asked after the first part of the experiment whether they really used mental rotation and whether they needed and were able to repeat the operation during the five seconds of stimulus presentation. According to their self-reports, no difficulties were encountered in following these instructions. Subjects did not report any discomfort associated with or negative feelings about TMS that could have otherwise influenced the results. The mean motor threshold (RMT) was 53.3% (SD 7.15) of the maximum stimulator output intensity; hence mean stimulation intensity for the experiment was 63.9% (SD 8.62). There was no difference between the baseline MEP amplitudes recorded before and after the mental rotation conditions as tested by a Wilcoxon test (*z *= 2.61; *p *= 0.79). Therefore, we refer to baseline MEP amplitudes as the mean MEP amplitudes for both baseline sessions. The baseline MEP amplitudes were then compared with the mean MEP amplitudes of the five mental rotation conditions, using a Friedman-ANOVA with five stimulus categories and the baseline condition as within-subject factors. We included all 20 MEP amplitudes for analysis because the results were no different when we excluded the data of the four TMS pulses in each condition that were delivered at the moment of the switch between trials. In any case, it was very unlikely that potential excitability of M1 would suddenly drop for this single moment even though there was a very short break between the mental rotations performed. The Friedman-ANOVA revealed a highly significant difference [*χ*^2^(5) = 42.91; *p *< .001]. Post hoc tests corrected for multiple comparisons revealed significantly stronger MEP amplitudes during mental rotation of four object categories compared with baseline (Shepard & Metzler figures *z *= 2.45, *p *< .001; houses *z *= 2.6, *p *< .001; tools *z *= 3.0, *p *< .001; 2-D figures *z *= 2.8, *p *< .001). The increased strength of MEP amplitudes during the mental rotation of hand stimuli compared with baseline did not reach significance (*z *= 0.85, *p *> .05). Looking at comparisons across object categories, MEP amplitudes were consistently and significantly smaller with the hand stimuli as compared with images of houses (*z *= -1.75, *p *< .05), tools (*z *= -2.15, *p *< .01) and 2-D figures (*z *= -1.95, *p *< .05) as well as tending to be smaller compared with Shepard & Metzler figures (*z *= -1.60, *p *> .05) (see Fig. 3).

For the second part of the experiment, an ANOVA for repeated measurements (again using the five stimulus categories as within-subject factors) showed that the RTs differed for mental rotation of the different object categories [*F*(4,76) = 78.64; *p *< .001; *R*^2 ^= 0.69]. RTs were included only for trials in which subjects answered correctly (see Table 1). Post hoc Tukey tests corrected for multiple comparisons revealed that RTs were longer for Shepard & Metzler figures (*p *< .001) as well as for houses (*p *< .001) compared with tools, which in turn showed longer RTs compared with hands (*p *< .001) and 2-D figures (*p *< .001). The correlations between MEP amplitudes and RTs were analyzed using Spearman's Rho. There was no significant correlation for any category of objects (Shepard & Metzler figures: *r *= -.25, *p *= .30; houses: *r *= -.28, *p *= .23; tools: *r *= -.18, *p *= .46; hands: *r *= -.08, *p *= .73; 2-D figures: *r *= -.14, *p *= .57). Correlations between MEP amplitudes and the numbers of errors were not significant either (Shepard & Metzler figures: *r *= -.15, *p *= .54; houses: *r *= .17, *p *= .48; tools: *r *= -.09, *p *= .71; hands: *r *= .24, *p *= .31; 2-D figures: *r *= .23, *p *= .33). The forced-choice ranking of difficulty assessed after the experiment was consistent with reaction times and errors (Table 1). Mental rotation of Shepard & Metzler figures were perceived as being most difficult, followed by houses, tools, hands and 2-D figures. Again, correlations between MEP amplitudes and the mean subjective ranking of difficulty did not reach significance in any of the categories (Shepard & Metzler figures: *r *= -.18, *p *= .45; houses: *r *= .05, *p *= .83; tools: *r *= .41, *p *= .07; hands: *r *= -.33, *p *= .16; 2-D figures: *r *= .01, *p *= .98). Finally, we used Wilcoxon tests to compare ratings of strategy frequency for each category of objects. In all categories, subjects indicated the use of the external strategy more often than the use of the internal strategy (see Table 1). This difference was highly significant for all stimulus categories except for hands for which the effect was less pronounced (Shepard & Metzler figures: *z *= 3.57, *p *< .001; houses: *z *= 3.55, *p *< .001; tools: *z *= 3.25, *p *< .001; 2-D figures: *z *= 3.36, *p *< .001; hands: *z *= 1.75, *p *= .08).

**Table 1:** Descriptive statistics for n = 20 subjects.

	Shepard & Metzler fig.	Houses	Tools	Hands	2-D figures	Mean baseline
Mean MEP amplitudes [μV]	732.64	731.47	785.21	629.37	802.30	496.65
SD MEP amplitudes [μV]	518.52	535.86	563.90	521.31	613.79	361.70
Mean reaction times [ms]	3115	3217	2381	1869	1981	-
SD reaction times [ms]	428.24	280.50	449.62	439.97	304.97	-
Mean number of errors	7.75	5.15	2.55	0.95	0.80	-
SD number of errors	3.06	2.72	1.85	1.23	0.89	-
Mean ranking difficulty	4.70	4.20	2.70	1.75	1.65	-
SD ranking difficulty	0.57	0.52	0.80	0.72	0.75	-
Frequency use of internal strategy	0.50	0.40	0.75	1.40	0.50	-
SD use of internal strategy	1.05	1.00	1.33	1.70	1.28	-
Frequency use of external strategy	3.45	3.50	3.25	2.75	3.50	-
SD use of external strategy	1.10	1.00	1.25	1.65	1.24	-

MEP = motor evoked potentials; SD = standard deviation; recording of MEP amplitudes in first part, reaction times and errors in the second part of the experiment; ranking difficulty was forced-choice ranking: from 5 = "hardest" to 1 = "easiest"; frequency use of strategy refers to internal and external strategies described by Kosslyn et al. [3] assessed by post hoc rating questionnaire from 0 = "never used" to 4 = "always used" (the mean rating is reported here).

## Discussion

Our data show that single TMS pulses applied to the left primary motor cortex evoke stronger MEP amplitudes in the contralateral APB muscle during mental rotation as compared with a resting baseline. This holds for all categories of rotated objects except hands. Furthermore, the extent of the MEP increase is similar for the rotation of 3-D Shepard & Metzler figures, houses, tools and 2-D figures. This finding is in line with previous results from our lab that additionally showed the exclusiveness of increased MEP amplitudes for mental rotation compared with several control conditions [17].

The strategy hypothesis [3,6] predicted that pictures of hands as well as tools are more likely to trigger the use of an internal strategy. This is based on the proposition that it is easier to imagine rotating ones own hands or tools. The use of such a body-related rotation strategy is supposed to cause primary motor cortex involvement and should therefore lead to higher corticospinal excitability as reflected by higher MEP amplitudes. On the other hand, we hypothesized that the abstract 3-D Shepard & Metzler figures, 2-D figures as well as pictures of houses would not cause M1 activation, because these stimuli would be more likely to trigger the external strategy: Subjects would imagine the objects being rotated by external forces. However, our results do not support this hypothesis. We did not find a significant difference between the MEPs obtained during mental rotation of the different figures.

One might argue that it is not the particular mental rotation strategy that modifies M1 activation but the difficulty of the mental rotation task. If task difficulty was the main reason, the increase in effort to mentally rotate objects would have been expected to cause the entire neural circuit to operate at a higher activation level compared with the less demanding task. However, we did not find significant correlations between task difficulty (indicated by RTs and error rates) and MEP amplitudes. Given that task difficulty is also a subjective experience, we also examined whether there is a relationship between subjective task difficulty and MEP amplitudes. Again, there was no relationship indicating the independence of MEP amplitudes and, thus, M1 activation (including activation of the corticospinal tract) from subjectively experienced task difficulty.

It could also be argued that subjects did not follow the instructions to use mental rotation for the task, since we did not examine the relationship between angle disparity between the objects and reaction times. This, however, is very unlikely because all subjects reported having used the general strategy of mental rotation during the experiment. They all completed the post-experimental questionnaire and agreed with at least one of the given descriptions of mental rotation as corresponding with their performed operation. Examining the post-experimental ratings of strategies used for mental rotation in more detail, we found that the subjects all but exclusively reported the use of an external strategy for Shepard & Metzler figures, houses, tools and 2-D figures, with the exception of the hand stimuli for which only a marginal difference was found between the frequency of used strategies. Even though the questionnaire additionally asked for other strategies not assed by the two given descriptions, none of the subjects indicated the use of a different strategy. Obviously, the so-called *external strategy *is the most common used strategy in our study more or less independent from the rotated objects.

Given that task difficulty as well as differences between strategies cannot explain why M1 and the adjacent corticospinal tract are activated during mental rotation, what else could be a satisfying explanation? In some way, this study confirms the conclusions from Eisenegger et al. [17] that mental rotation itself causes the excitability of M1. One possibility is that M1 is directly involved in mental rotation. Results of Georgopoulos et al. [24] demonstrated the existence of direction-sensitive neurons in M1. These neurons could also play a role in planning and imagining of the mental rotation process. Single pulse TMS applied over the hand as well as the foot area of M1 showed that simply listening to sentences involving hand and foot actions modulated MEP amplitudes measured at the corresponding muscles, respectively [25]. MEP amplitudes evoked by TMS applied to M1 were also modulated by only visualizing motor actions without acting them out [26], and performance in mental rotation of body parts could be disturbed by TMS and intra-cortical stimulation [14-16]. These findings point towards a direct involvement of M1 in imagining the rotation process but could also be restricted to body parts and depend on the exact task.

The other possibility is that the strong excitability of M1 during mental rotation simply reflects a spill-over effect from adjacent and strongly activated brain regions [6,17]. Based on findings demonstrating that posterior parietal cortex and premotor cortex are activated in mental rotation, it was concluded that these areas are involved in spatial transformations and operations [2,4,27-29]. From studies in monkeys and humans it is known that the lateral premotor cortex and M1 are densely interconnected both anatomically and functionally [30-36]. In addition, it has been shown that TMS-induced neural activity does show spill-over effects, even at a sub-threshold level [37-39]. Bestmann et al. [38] used simultaneous TMS and fMRI to show that rTMS applied to the primary motor cortex led to changes of the haemodynamic response not only in M1, but also in anatomically and functionally connected regions such as primary sensory cortex (S1), SMA, dPMC, Cingulum, Putamen and Thalamus. In a later paper of the same group, similar functional remote effects were reported for rTMS applied over the dPMC [39]. Altogether, these findings provide strong support for the hypothesis that task-inducted neural activity could also lead to spill-over effects. That there may be no causal involvement of M1 in mental rotation is therefore possible. This explanation would be in line with a brain imaging study that reported only premotor cortex but not M1 activation during mental rotation using motor imagery [40]. M1 may be excitable only because of the activation of premotor cortex. Under real-life conditions, many cognitive processes prepare for subsequent actions. Premotor cortex activation during the mental rotation of objects could therefore prepare subjects for the possibility of acting on them [6]. Such preparation must not be bound to the strategy used for mental rotation; it could be executed for all objects generally. Following this argument, pictures of hands might represent an exception since they do not require the preparation of object-related, manipulative actions. Subjects may have simply perceived them as body parts, thus rendering a preparatory response to act on them unnecessary. One can speculate that premotor cortex activation for hands did not have to be as strong as for the mental rotation of the other objects, especially since subjects indicated that they did not make predominant use of a strategy that involved direct motor imagery, and that the over-spilling activation to M1 as well as the excitability of M1 was therefore comparably smaller. Another explanation is that visualizing a rotating body part (e.g. a hand) led to inhibition of subjects' action schemas and therefore to decreased MEP amplitudes, such as observed when subjects listen to sentences involving action [25], and that this may have occurred because the instructions required subjects not to act during the TMS session. However, finding a compelling explanation for the differences between objects and body parts is beyond the focus of this study and should be the target of more specific and systematic studies in the future.

## Conclusion

This study demonstrates that the primary hand motor area and the associated corticospinal tract are strongly activated during mental rotation of different objects. This activation is present even when no explicit motor task is required. In addition, we could not corroborate the hypothesis that only rotating pictures of hands or tools would implicitly activate the primary hand motor area. Instead, we found a general and unspecific activation increase that might be due to a "spill-over" effect from adjacent brain regions.

## Abbreviations

APB, Abductor Pollicis Brevis;

EMG, electromyogram;

dPMC, dorsal premotor cortex;

ISI, inter-stimulus interval;

M1, primary motor cortex;

MEP, Motor Evoked Potential;

RMT, resting motor threshold;

RT, reaction time;

S1, primary sensory cortex;

SD, standard deviation;

SE, standard error;

SMA, supplementary motor area;

TMS, Transcranial Magnetic Stimulation;

## Competing interests

The author(s) declare that they have no competing interests.

## Authors' contributions

LJ and SB designed the study. SB carried out the study realization, performed the statistical analysis and drafted the manuscript. SK substantially contributed to the study realization. SB, SK and LJ have also been involved in interpreting the data, drafting the manuscript and revisiting it. All authors read and approved the final manuscript.
